# The association between armed conflict, violence and mental health: a cross sectional study comparing two populations in Cundinamarca department, Colombia

**DOI:** 10.1186/1752-1505-6-12

**Published:** 2012-12-17

**Authors:** Alicia Londoño, Perla Romero, Germán Casas

**Affiliations:** 1 Los Andes University School of Medicine, Bogota, Colombia

**Keywords:** Armed conflict, Violence, Colombia, Guasca, Guatavita, Mental health

## Abstract

**Background:**

Exposure to violence in general and to armed conflict in particular has been consistently associated with an increased prevalence of mental illness. Colombia has sustained an internal armed conflict for decades and is considered one of the most violent countries in the world. However, certain areas have been more exposed to the conflict than others.

**Methods:**

This is a cross sectional study comparing two communities from different villages in the department of Cundinamarca, Colombia. One, Guasca, was directly impacted by armed conflict. The other one; Guatavita has never been affected by armed conflict. We applied two different instruments: the PHQ scale and a short standardized interview in order to estimate the prevalence of major psychiatric disorders and their link to violent events. Forty-two volunteers from each village were evaluated through a personal interview using these two instruments.

**Findings:**

Of the population surveyed in Guatavita, 2.4% reported direct exposure to violence compared to 23.8% from Guasca. In the population exposed directly to violent events, the prevalence of all disorders was greater than in the non-exposed population with an OR of 1.46 (95% CI 0.3809 - 5.5989) for anxiety; 4.54 (95% CI 1.1098 - 18.5984) for depression; 6.0 (95% CI 1.2298 - 30.2263) for somatization disorder; and 4.4 (95% CI 1.2037 - 16.0842) for alcohol abuse.

**Interpretation:**

There is a statistically significant association between the history of armed conflict, violence and the presence of mental illnesses, particularly depression, somatization disorder and alcohol abuse. Special attention should be paid to the detection, prevention and treatment of these disorders when dealing with populations exposed to violence and to armed conflict in particular.

## Background

Colombia is the only country in the Western hemisphere that has sustained an armed conflict that predates the emergence of Marxist guerrilla groups in Latin America in the early 1960s. The World Health Organization has listed Colombia as one of the most violent countries in the world for the past decades having the 4th highest armed conflict related deaths worldwide for the period between 2003 and 2007 [1]. According to statistics from the Colombian Institute of Forensic Medicine (ICML), the majority of victims and perpetrators of violence in the country are between the ages of 15 and 35 years [2-8].

**Figure 1:**
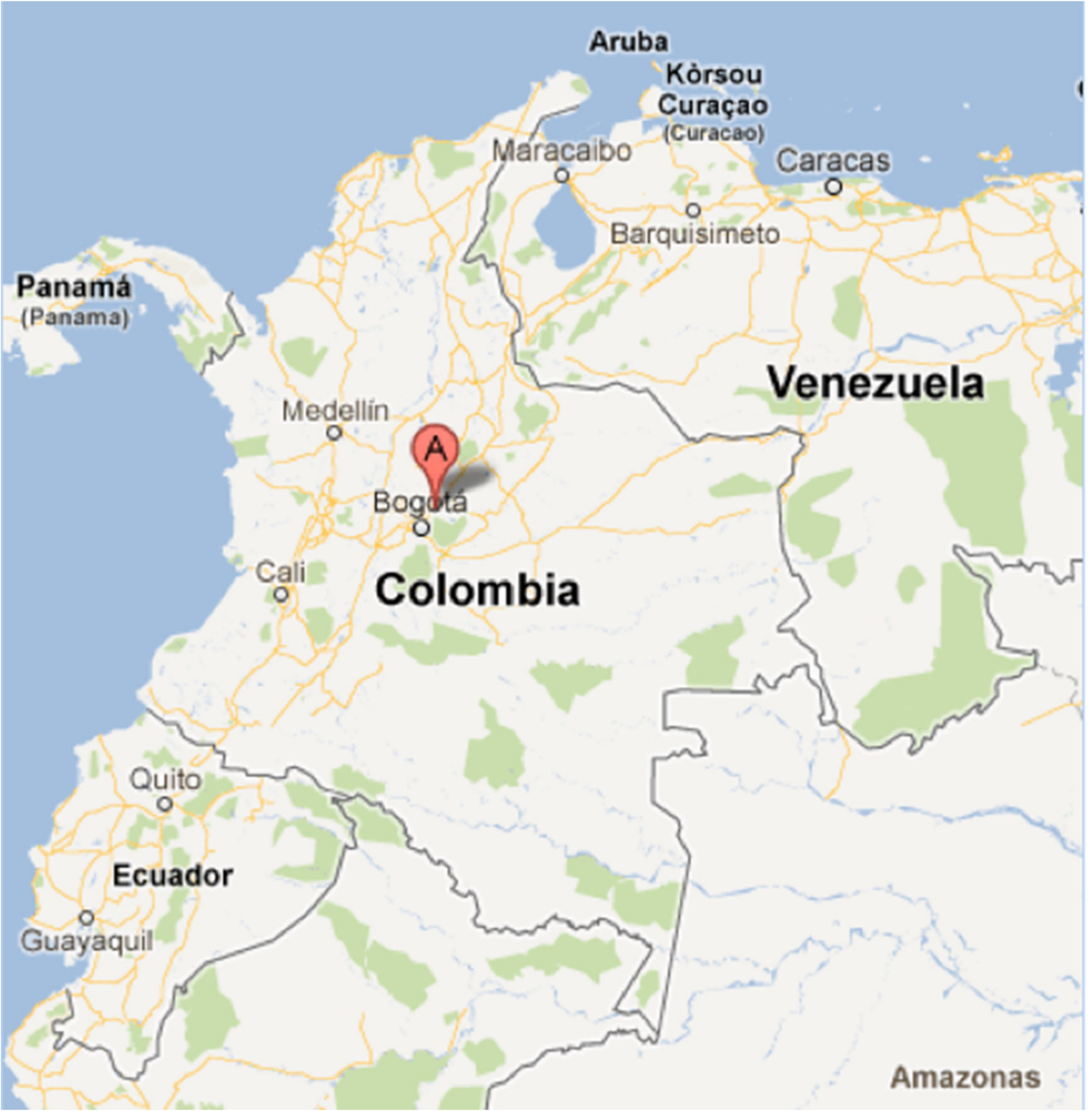
**Map of Colombia indicating where Guasca and Guatavita are located.** Image taken from google maps. http://maps.google.es/.

**Figure 2:**
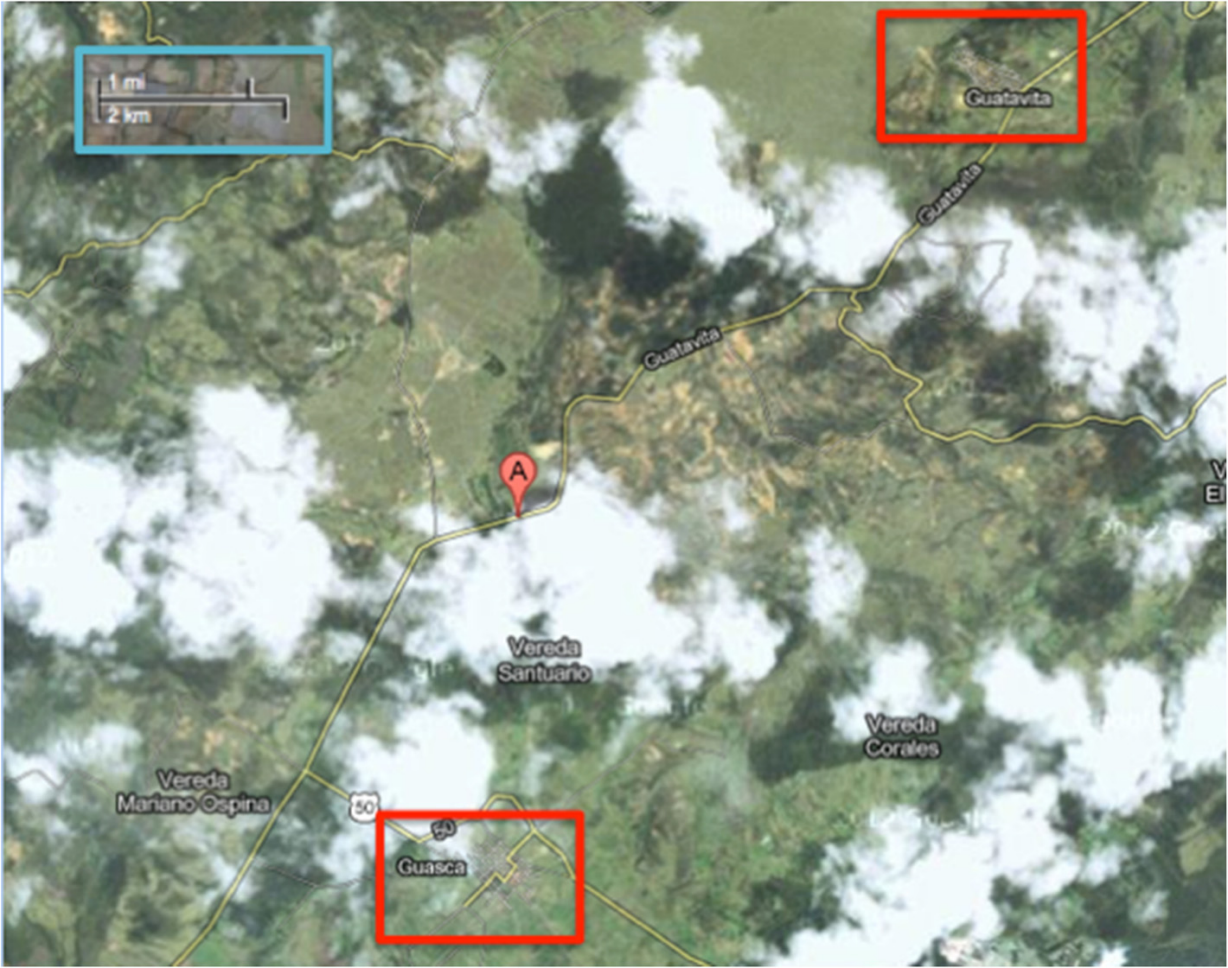
**Satellite map showing Guasca and Guatavita in close proximity to one another.** Image taken from google maps. http://maps.google.es/.

This conflict has affected rural areas disproportionately. In the early of the 1990s, there was a significant increase in the presence of armed groups in several villages near Bogota, in the department of Cundinamarca [9]. Guasca, a village in Cundinamarca (Figures 1 and 2) was heavily affected by armed conflict in the mid 1990s. The reason for Guasca’s involvement in armed conflict was its convenient geographical position, located between a guerilla camp in the country’s central mountain range and the Bogota savanna. Although conflict in this area has largely subsided, it continues to be one of the most violent villages in the region, with a homicide rate of 29.99 per 100,000 people in 2010 [2].

On the other hand, Guatavita (Figures 1 and 2), a village that borders Guasca on the north has never sustained armed conflict nor has it ever been particularly violent, having had no homicides in the year 2009 [10] and 14.93 per 100,000 people in the year 2010 [2]. Apart from this aspect, Guasca and Guatavita are very similar in terms of demographic, economic and social variables.

The prolonged armed conflict within our country has meant that many civilians have been exposed to violent events from early ages. This is particularly worrisome because one of the well-known risk factors known to trigger psychiatric morbidity is living in a conflictive environment. Trauma studies worldwide have shown a short- and long-term higher prevalence of mental health disorders including depression, anxiety, somatization disorders and drug abuse in populations exposed to violence [11-14].

In his article “Child soldiers: understanding the context” [12] Professor Daya Somasundaram comments that among 625 war-exposed adolescents, 31% were diagnosed with Post Traumatic Stress Disorder, 32% with somatization disorder, 34% with anxiety and 29% with depression, among other mental illnesses.

The study “Trauma-related psychological disorders among Palestinian children and adults in Gaza and West Bank” by Emmanuelle Espié et al [13]. also illustrates the effects of violence on mental health, showing that among 1,253 patients attending psychological support services in the West Bank and Gaza Strip, 23% presented PTSD, 17.3% anxiety disorders, and 15.3% depression. This study also showed different mental health disorders in populations that had been chronically affected by trauma indicating that prolonged trauma related to armed conflict has different consequences on mental health than acute traumatic events.

We believe the lack of early identification and treatment of mental disorders in populations that have been chronically exposed to armed conflict related trauma, such as Colombia, can hinder national efforts to promote human development. In this study we aimed to evaluate the association between violence and mental health in regions exposed to armed conflict in Colombia.

## Methods

This was a cross sectional study comparing the mental welfare of young adults from the villages of Guasca and Guatavita. We asked young citizens in the streets of Guasca and Guatavita for their collaboration. We interviewed literate residents between the ages of 20 and 30 who had lived in their villages for at least ten years. Exclusion criteria included any psychotic disorder, mental retardation, sensorial, or language limitation or current cognitive symptomatology that impeded the interview. We targeted this age group because the country’s forensic medicine institute has documented it has bore the brunt of the conflict.

### Instruments

We used two instruments for data collection. The first one is the PHQ scale, which is a self-administered questionnaire consisting of multiple-choice questions and is commonly used as a tool for screening for depression, anxiety, alcohol abuse, eating disorders and somatization disorders in primary care [15-18]. We followed the guidelines suggested by the PHQ manual for the interpretation of the results, using a cut-point of 5 points or more to diagnose mild depression, 10 points or more to diagnose moderate depression and 15 points or more to diagnose severe depression with the PHQ-9 section of the PHQ scale. We used a score of 5 or greater in the GAD-7 subscale to diagnose anxiety and a score of 5 or greater and 10 or greater in the PHQ-15 subscale to diagnose mild and moderate somatization disorder, respectively. We also followed the recommended criteria for the diagnosis of eating disorders and alcohol abuse, making a diagnosis of an eating disorder with affirmative answers to the first three questions in this section and making a diagnosis of alcohol abuse for an affirmative answer to any of the questions in that section.

We also developed a short questionnaire to evaluate socio-demographic variables and exposure to violent events. This brief questionnaire documented gender, age, occupation, educational level, number of people they lived with, medical illnesses and military service. For legal reasons we did not inquire about participation in other armed groups. It also tracked exposure to violent events (described as physical abuse by someone external to their family, physical abuse by someone in their family and rape).

### Location and timing

During the months of September and October of the year 2011, two final year medical students from Los Andes University applied both instruments in the villages of Guasca and Guatavita, Cundinamarca. Volunteers were sought in the main streets of both villages on weekends, where we explained the intent of our research and the methodology of our study. About 70% of the people we approached in Guasca and 90% in Guatavita agreed to participate.

### Sample size

According to statistics from the National Administrative Department of Demographics of Colombia (DANE), there were a total of 784 and 2148 adults between the ages of 20 and 30 in Guatavita and Guasca respectively in 2010. Forty-two subjects from each town would be needed to detect a RR=2.0 with 80% power and 5% probability of type I error.

### Ethical considerations

This study follows the conditions established by the Colombian Government and has been qualified as a non risk study according to resolution 8430 which establishes scientific, technical and administrative norms. The study’s design and ethical risks were validated according to this law by one of the ethical and scientific authorities at Los Andes University School of medicine.

Each participant was asked for verbal consent. We explained to each volunteer that participation would be kept anonymous and confidential. After interviews were completed participants were asked to place the interviews in a sealed box. Names and other identification details were not documented in order to encourage participation and honest answers.

We emphasize that the diagnostic tools used in our study are designed primarily for population screening and are therefore limited. Local health authorities were informed of our results in order to provide proper diagnostic strategies that confirm our findings and to outline treatment and prevention plans accordingly.

### Quantitative data analysis

Tabular data was first entered into an Excel spreadsheet (Excel for Mac version 14.0.1-101012) and analyzed using SAS (9.2). We calculated the prevalence of each disorder for the population exposed to violence versus the non-exposed. Relative Risks were estimated based on Odds Ratios (OR) looking for associations between exposure to violent events and mental illness symptomatology.

## Results

### Demographic variables

Forty-two volunteers from Guatavita and forty-two volunteers from Guasca participated in the study by completing both the PHQ scale and the socio-demographic variable interview. The mean age was 24 years of age in Guasca and 23.5 in Guatavita. Of the sampled population, 25.19% were male and 74.81% were female in Guatavita while in Guasca 26.66% were male and 73.34% were female. Most of the participants in both towns were students and declared their educational level as “technician”. Most lived with their mother or both parents and siblings. Only one of the participants in Guasca and none in Guatavita served the army.

### Exposure to violence

2.4% of the volunteers in Guatavita and 23.8% from Guasca reported having been directly exposed to some kind of physical violence (physical abuse by someone in their family, physical abuse by someone external to their family or rape) with an odds ratio of 12.8 (95% CI of 1.558 - 105.3655). Most of the violent events reported were physical abuse by someone in their family followed by physical abuse by someone external to their family and there was only one case of rape reported.

### Comparison of prevalence of mental illness symptomatology in Guasca and Guatavita

The prevalence of anxiety, somatization disorder, alcohol abuse and eating disorders was greater in Guasca than Guatavita with a prevalence of 32.5% and 25.7% for anxiety, 73.8% and 61% for somatization disorder, 11.9% and 4.7% for eating disorders and 38.1% and 23.8% for alcohol abuse. The score for depression was very similar in both villages with a prevalence of 47% in Guasca and 50% in Guatavita. However, none of these differences were statistically significant.

When comparing the population exposed directly to the violent events inquired in the socio-demographic variable interview in both villages with the non exposed population, the prevalence of anxiety, depression, somatization disorder and eating disorders was higher in the first group (Table 1) with an OR of 1.46 (95% CI 0.3809 - 5.5989) for anxiety, 4.54 (95% CI 1.1098 - 18.5984) for depression, 6.09 (95% CI 1.2298 - 30.2263) for somatization disorders, 1.30 (0.1416 - 11.9342) for eating disorder and 4.40 (95% CI 1.2037 - 16.0842) for alcohol abuse.

**Table 1:** Comparison of prevalence and severity of mental illness symptomatology in population directly exposed to violence vs. non-exposed in both villages

**Mental health condition**	**Type of data**	**Direct exposure to violence**	**No direct exposure to violence**
**Anxiety (N =75)**	Positive score (≥5 in GAD-7) n/N (%)	4/11(36.36%)	18/64 (28.12%)
	Positive score for moderate to severe anxiety (≥10 in GAD-7) n/N (%)	2/11 (18.18%)	1/64 (1.56%)
	Mean GAD-7 score	4	2.57
**Depression (N=84)**	Positive score (≥5 in PHQ-9) n/N (%)	8/11 (72.72%)	27/73 (32.14%)
	Positive score for moderate to severe depression (≥10 in PHQ-9) n/N (%)	3/11 (27.27%)	4/73 (4.76%)
	Mean PHQ-9 score	7.45	4.26
**Somatization Disorders (N=84)**	Positive score (≥5 in PHQ-15) n/N (%)	9/11 (81.81%)	31/73 (37.8%)
	Positive score for moderate to severe Somatization Disorders (≥10 in PHQ-15) n/N (%)	7/11 (63.63%)	8/73 (9.52%)
	Mean PHQ-15 score	10.45	5.8
**Eating disorders (N=84)**	Positive result n/N (%)	1/11 (9.09%)	6/73 (7.14%)
	Negative result n/N (%)	10/11 (90.9%)	78/73 (92.85%)
**Alcohol abuse**	Positive result n/N (%)	6/11 (54.54%)	18/73 (21.42%)
	Negative result n/N (%)	5/11 (21.42%)	66/73 (78.57%)

## Discussion

The prevalence of exposure to violence was ten times greater in Guasca compared to Guatavita (2.4% vs. 23.8%) with an OR of 12.8 (95% CI of 1.558 - 105.3655). This is consistent with what the statistics and historical record indicate [2]. Although not currently afflicted by armed conflict as it once was, the fact that the prevalence of violent events continues to be much higher in Guasca than in Guatavita likely represents a consequence of the history of armed conflict in this village, and speaks of the extensive and trans-generational effects that armed conflict can leave behind.

The results shown in table two suggest that in Colombia, young people exposed to violence are more likely than those who are not exposed to present psychiatric symptomatology, corroborating what studies in other parts of the world have shown: there is a link between exposure to violence and mental illness [14,19-21].

Only the observed differences for depression, somatization disorder, and alcohol abuse were statistically significant, with somatization disorder followed by depression and alcohol abuse having the greatest strength of association to violence exposure (Table 2). Although the observed differences for general anxiety disorder and eating disorders were not statistically significant, they do follow this same trend.

**Table 2:** Strength of association between exposure to violence and psychiatric symptomatology

**Mental illness**	**Odds ratio**	**95% Confidence interval**	**P value**
**Anxiety**	1.46	0.3809 - 5.5989	0.40
**Depression**	4.54	1.1098 - 18.5984	0.02
**Somatization disorder**	6.09	1.2298 - 30.2263	0.01
**Eating Disorder**	1.30	0.1416 - 11.9342	0.64
**Alcohol Abuse**	4.40	1.2037 - 16.0842	0.05

The prevalence of somatization disorder and depression were particularly high in the population exposed to violence (81.81% and 72.72% respectively), much higher than what other studies in different countries and scenarios have found [12,13].

The prevalence of mental health disorders estimated in Guatavita, where there is little direct exposure to violence is still high compared to other countries such as The United States of America and Canada [18]. This might be related to several factors including socio-demographic, cultural or economic differences. However it might also be related to the fact that even though Guatavita has not endured an armed conflict like Guasca, it is still part of a violent country and region and its population might still be indirectly affected by violence, which could have a detrimental effect even on individuals living outside particularly violent zones.

Finally, the fact that more people were willing to participate in Guatavita than Guasca might allude to the cultural background of each village as well as suggest that people may be less willing to discuss exposure to violence in places more heavily affected by it. Also, women were oversampled in both towns firstly because in general they where more open to volunteering in the study and also due to the fact that there were more women in the main town plaza where the participants were sought while men were more likely to be working in the farms around the villages.

### Limitations

These results show an association between violence, armed conflict and mental illness. However it is important to state they cannot determine causality. It is also important to recognize these results show a trend in mental health but cannot be considered diagnoses of the disorders in question because the instrument used (PHQ scale) is not intended for this propose. Also, careful consideration must be taken when comparing these results with the results from other studies in different countries given that different evaluation strategies have been used and the type of violence exposure can vary.

It is important to acknowledge that violence can take many forms, ranging from physical attacks to emotional abuse. In this study we only evaluated “physical” violence that were easier to describe and evaluate with a short questionnaire (inquiring for physical and sexual abuse). It is likely that other types of violence such as emotional and verbal abuse were present in both villages and could also have an impact on mental health.

The sample size used was small in comparison to the overall population in the villages. This is reflected in the wide range of the 95% CI we obtained. We emphasize the need to conduct larger studies in order to validate our results and acquire greater insight into the subject.

Also, the fact that all of the participants were volunteers that were approached on the streets represents a sample bias, and it must be taken into account that those staying at home or not stopping to answer questions could be different to the participants in this study. Also, the fact that the majority of the participants were women must be taken into consideration when interpreting the results.

Finally, although it is beyond the scope of this article, it is important to recognize that the Colombian conflict has resulted in a large number of displaced people, particularly from areas most affected by armed conflict. It is possible that those who were most affected (and therefore more likely to have psychological scars) have left the villages (particularly Guasca) being unavailable for interviews. Although this would represent a downward bias it is important to mention. It would also be interesting to corroborate these results with different age groups.

## Conclusions

This study is an affirmation of the negative effects armed conflict and its violence aftermath can have on the mental health of individuals. It is pivotal in a country like Colombia, which has had a terrible history of armed conflict and violence to emphasize the importance of the early recognition and treatment of mental illness, given that unfortunately most of the time the distress of these individuals goes unheeded. The early identification and treatment of mental illness in populations affected by armed conflict should be fundamental pillars of public health politics, not only for the well-being of citizens but also as a necessary step in the progress and development of the nation as a whole.

## Competing interests

We declare that we have no conflict of interest.

## Authors’ contributions

AL designed the study; oversaw the collection of the data; conducted the quantitative data analysis, and wrote the first draft of the paper. GC provided guidance in the analysis and interpretation of results and in the writing of the paper, reviewed the qualitative data analysis write-up and assisted in writing the quantitative analysis section of the paper. PR helped design the interview and aided with the administration of the study. All authors criticized drafts of the paper. All authors read and approved the final manuscript.
